# Molecular epidemiology and clinical features of hand, foot and mouth disease in northern Thailand in 2016: a prospective cohort study

**DOI:** 10.1186/s12879-018-3560-4

**Published:** 2018-12-06

**Authors:** Panupong Upala, Tawatchai Apidechkul, Wipob Suttana, Niwed Kullawong, Ratipark Tamornpark, Chadaporn Inta

**Affiliations:** 1 Center of Excellence for the Hill-tribe Health Research, Mae Fah Luang University, 333 Mo.1 Tasud Subdistrict, Muang District, Chiang Rai, Chiang Rai Province 57100 Thailand; 2 School of Health Science Research, Mae Fah Luang University, 333 Mo.1 Tasud Subdistrict, Muang District, Chiang Rai, Chiang Rai Province 57100 Thailand

**Keywords:** Hand, Foot and mouth, Molecular epidemiology, Clinical feature, Serotype, EV-A71, Coxsackievirus

## Abstract

**Background:**

Hand, foot and mouth disease (HFMD) is a major communicable disease in children ≤6 years old, particularly in several countries in the Asia-Pacific Region, including Thailand. HFMD impacts public health and the economy, especially in northern Thailand.

**Methods:**

A prospective cohort study was conducted to estimate the incidence rate and to identify the serotype and clinical features of HFMD among children in northern Thailand. A validated questionnaire and throat swab were used for data collection. Polymerase chain reaction (PCR) was used to detect human enterovirus and identify its serotypes. Participants were recruited from 14 hospitals in two provinces in northern Thailand, specifically, Chiang Rai and Pha Yao Province, between January 1, 2016, and December 31, 2016. Chi-square or Fisher’s exact test was used to detect the associations of signs and symptoms with HFMD serotype. Logistic regression was used to detect the associations of variables with a positive enterovirus at alpha = 0.05.

**Result:**

In total, 612 children aged ≤6 years from Chiang Rai and Pha Yao Province who were diagnosed with HFMD by a throat swab were recruited for the analysis. Approximately half of the cohort was male (57.2%), 57.5% was aged < 2 years, and 57.5% lived in rural areas. The incidence rate was 279.72/100,000 person-years in Chiang Rai Province and 321.24 per 100,000 person-years in Pha Yao Province. Additionally, 42.5% of children were positive for human enterovirus; among these children, 56.1% were positive for enterovirus-A (EV-A), 17.7% were positive for coxsackievirus (CV), and 26.2% were positive for other human RNA enteroviruses. During the study period, 21 distinct outbreaks of HFMD were recognized. Four to five patients (total 92 patients) were selected from each outbreak for identifying its serotype; enterovirus-A71 (EV-A71) was detected in 34.8% of HFMD cases, coxsackievirus-A16 (CV-A16) in 26.1%, coxsackivirus-A6 (CV-A6) in 15.2%, coxsackievirus-A10 (CV-A10) in 10.9%, coxsackievirus-A4 (CV-A4) in 2.2%, coxsackievirus-B2 (CV-B2) in 2.2%, human rhinovirus in 2.2%, and unknown serotype in 6.4%. Multivariable analysis demonstrated that a history of breastfeeding for ≤6 months was associated with a higher chance of enterovirus infection than a history of breastfeeding > 6 months, and children who had mother who worked as farmers, daily wage employees, and unprofessional skilled jobs had a greater chance of enterovirus infection than those who had unemployed mothers. Coxsackievirus-infected children had a higher rate of rashes on the buttocks, knee, and elbow and fever but a lower rate of lethargy and malaise than EV-A71-infected children.

**Conclusions:**

EV-A71 is a major cause of HFMD in children < 6 years old in northern Thailand, but rash, fever, and mouth ulcers are mostly found in participants with coxsackievirus infection. Breastfeeding should be promoted during early childhood for at least 6 months to prevent HFMD particularly those mother who are working in unprofessional skill jobs.

**Electronic supplementary material:**

The online version of this article (10.1186/s12879-018-3560-4) contains supplementary material, which is available to authorized users.

## Background

Recently, hand, foot and mouth disease (HFMD) has been defined as a new public health threat with large-scale infections, especially in children who are living in the Asia-Pacific Region, including Thailand [1]. Personal hygiene, sanitation and meteorological factors, such as environmental temperature, wind speed, air pressure, relative humidity and rainfall amount, have been associated with epidemic diseases [2–5]. HFMD is caused by various human enteroviruses, such as enterovirus A71 (EV-A71), coxsackievirus A16 (CV-A16), CV-A6, and CV-A10. Different serotypes predominate in different regions [6–9] and present different severities of clinical signs and symptoms after disease onset, such as fever, reduced appetite, sore throat, painful sores in the mouth, a rash of flat red spots, headache, stiff neck, and death [10, 11]. The World Health Organization (WHO) reported that children aged < 6 years and living in crowded areas are a vulnerable population for HFMD. Various episodes of HFMD have been reported in different parts of the world [12]. There are limited treatment modalities for HFMD, but several licensed vaccines are available for EV-A71 [13–18].

Every year, a large number of HFMD cases are reported globally. In 2016, the WHO reported 69,121 cases of HFMD in Japan. In total, 2,468,174 cases were reported in China, with 220 deaths in 2016. Singapore reported 42,147 cases in 2016, and Vietnam reported 48,866 cases in the same year [19].

In 2017, the Bureau of Epidemiology at the Ministry of Public Health of Thailand reported that 70,377 patients (prevalence proportion was 107.57/100,000 population) were diagnosed with HFMD, including 3 deaths. The Ministry of Public Health also reported that the ratio of male to female patients with HFMD was 1:0.80, and 25.90% of those patients were less than one year old. Northern Thailand was recognized as having the highest prevalence of HFMD in Thailand at 129.06/100,000 population, while the overall national incidence rate of HFMD in 2016 was 78.46/100,000 person-years [20]. The number of HFMD cases varied according to the sex, age, and socioeconomic status of a population; there were more HFMD cases among males than among females, more patients with lower age than older age, and more patients with low economic status than high economic status [20]. Piyada et al. [21] reported that there were some differences in the serotype of HFMD in different regions of Thailand.

Northern Thailand is geographically unique; 70% of the areas are mountainous, with relatively low temperatures throughout the year compared to other regions of Thailand. Chiang Rai and Pha Yao provinces are located in the northernmost area of Thailand bordering Myanmar, Republic of Lao, and China. In 2017, approximately 1.3 million people lived in Chiang Rai Province, and 490,000 people lived in Pha Yao Province. Among these populations, 25–30% are classified as an ethnic minority. Approximately 85% of the population in the two provinces had access to health care under universal coverage schemes in which medical expenses are free of charge [22]. The remaining part of the population did not meet the requirements to access health care services free of charge because they did not have a national identification card (ID) card. In Thailand, a national identification card (ID) is required for free or subsidized government medical services. Approximately one-third of the population lives below the national poverty level [23–26]. Most ethnic minorities are recognized as low socioeconomic populations in Thailand [27]. Conditions such as geographical location, socioeconomic status and ethnicity were suspected to contribute to the different patterns of HFMD serotype in children and to impact the severity of clinical features of infected children. Most children were left in a day care center (DCC) during the day time [28, 29]. Inta et al. reported that the most vulnerable population for HFMD in northern Thailand was children who attended DCCs, and there are numerous outbreaks in DCCs during the early rainy season of the year [29]. Concerns about HFMD are related to not only treatment and care but also medical expenses through the health care system. Upala et al. reported that 7 USD (216 baht) and 115 USD (3678 baht) were spent per patient per visit for HFMD medical costs and diagnosis in outpatient departments (OPD) and inpatient departments (IPD), respectively [30].

**Fig. 1:**
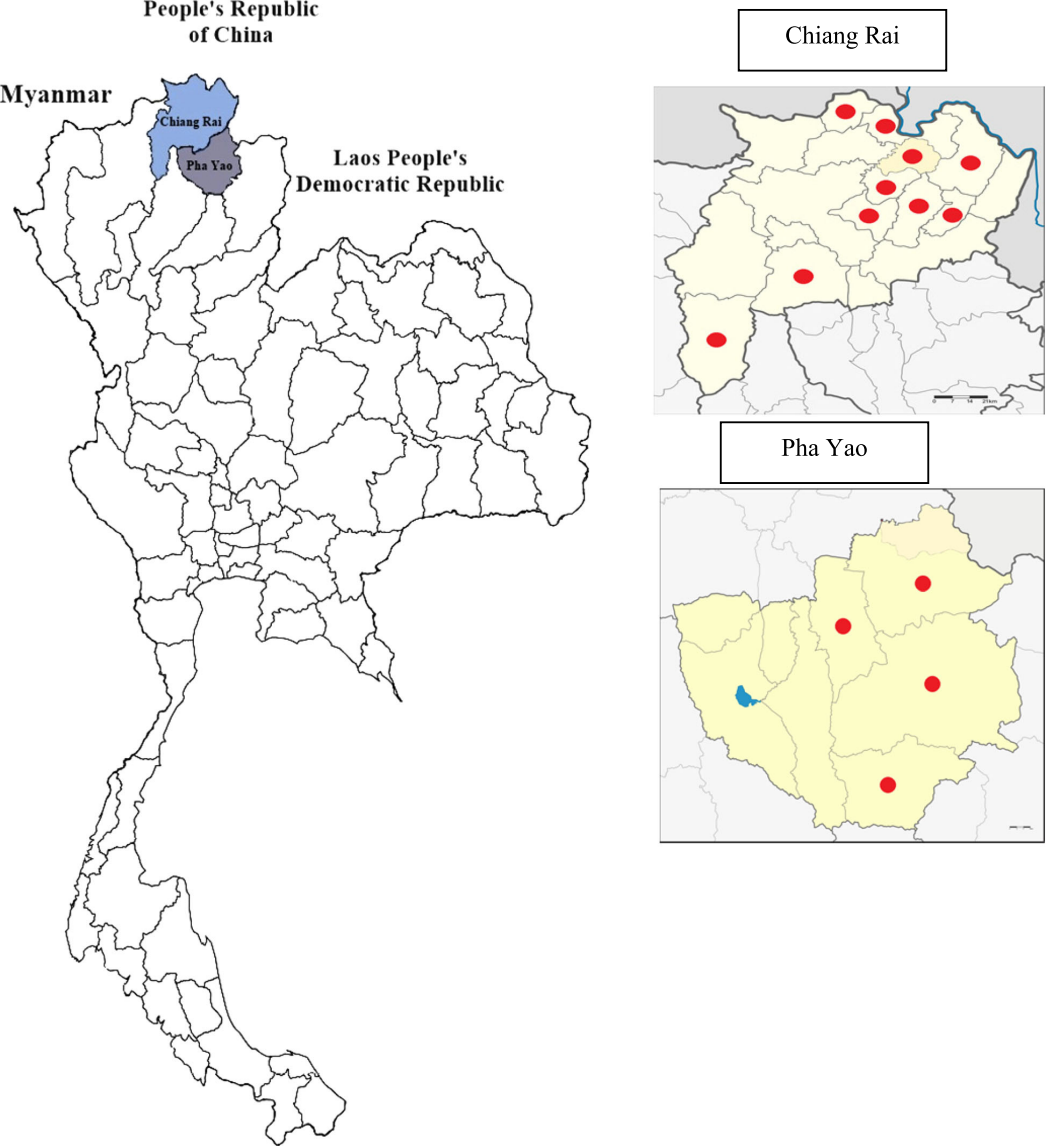
Map of the study areas and study settings in Chiang Rai and Pha Yao Province, northern Thailand. The red points show the participating hospitals

The objectives of this study were to conduct a comprehensive evaluation of HFMD epidemiology and to determine the molecular characteristics related to clinical features to test associations among a specific large-scale cohort in the highest epidemic areas, such as the northern areas of Thailand bordering Myanmar, Republic of Lao, and China (See Fig. 1).

## Methods

### Study design

A prospective cohort study was conducted to estimate the incidence rates, which were calculated by the number of new cases divided by the total number of children aged less than 6 years in the area for one year; to identify the dominant serotypes and their associations with clinical features; and to assess the risk factors for enteroviruses that cause HFMD among children less than 6 years old.

### Study sites

Data were collected from 14 hospitals in Chiang Rai and Pha Yao Province: 10 hospitals in Chiang Rai Province, and 4 hospitals in Pha Yao Province. In 2016, there were 16 hospitals in Chiang Rai Province and 6 hospitals in Pha Yao Province. However, the remaining 8 hospitals did not report HFMD cases during the study period.

### Study population

Children ≤6 years old living in Chiang Rai and Pha Yao provinces and diagnosed with HFMD between January 1, 2016, and December 31, 2016, from 14 hospitals where at least one case of HFMD was reported were included in the study.

### Study sample

All patients from each episode of an HFMD outbreak in Chiang Rai and Pha Yao provinces from whom a throat swab was obtained during the study period and in one of the 14 hospitals were selected as the study sample. An average of 10–15 patients in each outbreak were recruited. The first to the 10th–15th patient of each outbreak was recruited into the study with a purposive method which was based on the report at 10–15 HFMD cases in each outbreak in Thailand [31].

### Case definition

Children ≤6 years old attending one of the 14 hospitals in Chiang Rai and Pa Yao provinces who were diagnosed with HFMD by a medical doctor between January 1, 2016, and December 31, 2016, were included. All medical doctors in the study areas, including the 14 hospitals, used clinical signs under the International Classification of Diseases (ICD-10) system with code BP 08.4 [32] as the principle criteria for HFMD diagnosis.

#### Case diagnosis

HFMD diagnosis was performed using clinical signs and laboratory testing according to the standard protocol of the WHO, 2015 [1]. All medical doctors in Thailand were encouraged to use this standard guideline for HFMD case diagnosis and management.

#### Outbreak

An outbreak is the occurrence of more cases of a disease than would normally be expected in a specific place or group of people over a given period of time [33].

#### Outbreak detection

All public hospitals in Thailand have a surveillance system that networks between physicians and public health professionals, including epidemiologists. This system is operated by the Bureau of Epidemiology in the Ministry of Public Health [34]. The system is connected throughout the country. When an HFMD case was diagnosed by a physician, the diagnosis was sent to the epidemiology unit in the hospital. A case was then reported through the surveillance system network. The Epidemiology Department at the Provincial Public Health Office responds to the system and is alerted when there is an abnormal situation or when the mean number of HFMD cases is the mean of those in three previous years plus 2 standard deviation, which is the criteria for an outbreak and is commonly used in all healthcare settings in Thailand [34].

#### Case recruitment

In the first step, throat swabs were collected from 612 patients recruited into the study with an early diagnosis of HFMD by a medical doctor. All selected patients were asked to complete a questionnaire, and specimens were collected. In this step, the running number from the first case to the last case of each outbreak was labeled on both the questionnaires and tubes. The specimens were sent to the laboratory to identify human enteroviruses.

In the second step, 4–5 early cases in each outbreak labeled with running numbers from the first step that tested positive for human enterovirus species were selected to identify the serotype by a purposive method.

### Research instruments

A specific questionnaire was developed and used for data collection. There were 25 questions within 3 parts. In the first part, 14 questions were used to collect general information about the children, such as age; sex; weight; height; history of breast feeding; daytime care; congenital diseases, such as thalassemia, allergy, asthma, and G-6-PD (glucose-6-phosphate dehydrogenase deficiency); history of vaccination by the National Expanded Program of Immunization (EPI), as children who had been vaccinated were healthier than those who had not been vaccinated and were at lower risk of infection; group activity with other children in their village prior to infection; and history of receiving an HFMD diagnosis by a medical doctor. In the second part, 8 questions were used to collect the characteristics of parents and caregivers, such as marital status, occupation, education, and whether they were the major care giver. In rural Thailand, major caregivers are often not parents but could be a grandfather or grandmother. In the last part, 4 questions were used to collect information regarding the illness, such as the start date and end date of the illness, signs and symptoms, and details of changes during the course of the illness, referring to all signs and symptoms that changed from the start of the illness to the end of the illness.

The questionnaire (Additional file 1) was tested for reliability by the test-retest method. The validity of the questionnaire was determined by the item-objective congruence technique (IOC). A pilot study including 10 selected samples with characteristics similar to those of the target population was conducted before the questionnaire was used in the present study.

Sterile viral transport medium was used for specimen collection by trained doctors in each hospital. Throat swabs were obtained from participants. The virus serotype was detected using a molecular method at Chiang Rai Medical Science Laboratory Center.

### Specimen and data collection procedures

Access to hospitals was granted by the Chiang Rai and Pha Yao Provincial Public Health Officers. Hospital directors and relevant staff were asked to participate in the project. The methods of collecting specimens and completing the questionnaires were demonstrated to physicians, nurses, and medical technician staff who were working in the 14 hospitals before commencement of the project.

Once the outbreak was reported, the parents of all HFMD patients from outpatient (OPD) or inpatient (IPD) departments whose patients could provide a throat swab were invited to participate in the study. All invited parents were asked to provide written informed consent on behalf of their children. With consent from the parents, a physician filled out the questionnaire and performed a throat swab using the material provided, with assistance from a trained nurse or laboratory technician. Specimens were stored in a refrigerator at the hospital and were ready for transfer to the Chiang Rai Medical Laboratory Center for laboratory work the next day.

### Sample storage, transportation, and EV detection

**Fig. 2:**
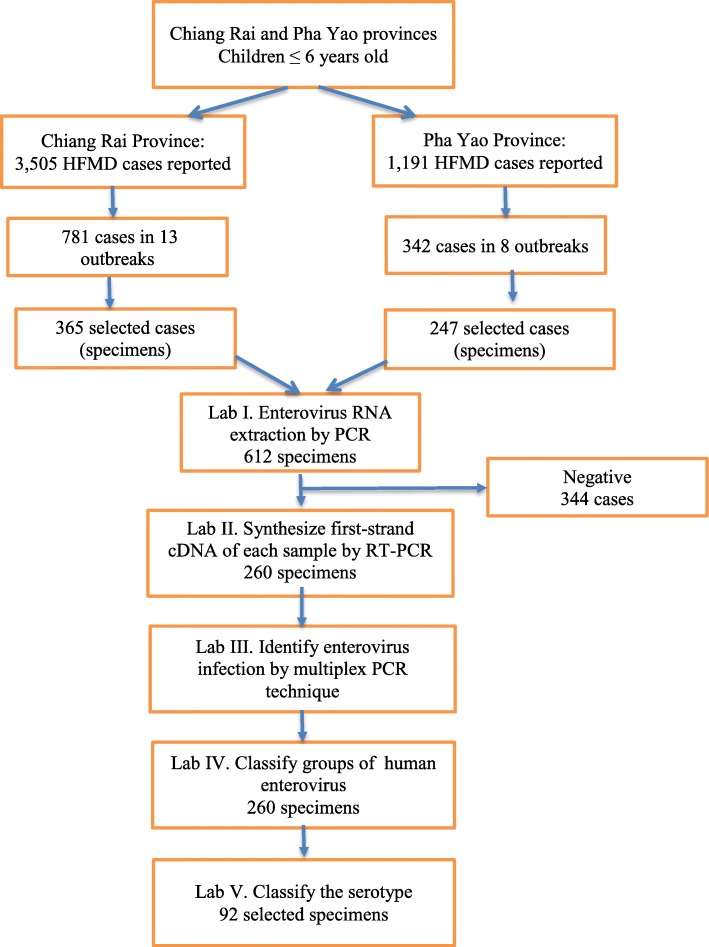
Steps of data collection and laboratory analysis from two settings; Chiang Rai and Pha Yao Province. In total, 4696 cases of HFMD from two Provinces were reported in 2016. 612 cases from 21 outbreaks were recruited into the study. Among them, 260 were positive for human enterovirus. 92 selected specimens were detected the serotyping

Specimens were stored in sterile viral transport medium and transported to the Chiang Rai Medical Science Laboratory Center the next day. The samples were stored until a sufficient number of samples for batch testing was reached. Below are the five steps for molecular genome identification of enterovirus and its subspecies (Fig. 2).

The first step was to extract enterovirus RNA from the specimens. Total RNA was extracted from all specimens using NucleoSpin® Virus (Pacific Science Co., LTD).

The second step was to synthesize first-strand cDNA for each sample. A volume of 5 μl of extracted total RNA was used as a template for first-strand cDNA synthesis (SuperScript™ III Reverse Transcriptase, Invitrogen) with 1X First-Strand buffer, conserved amino acid sequence-specific primers (AN32, 5’-GTY TGC CA-3′; AN33, 5’-GAY TGC CA-3′; AN34, 5’-CCR TCR TA-3′; AN35. 5’-RCT YTG CCA-3′), 10 mM DTT, 0.2 units/μl RNaseOUT™ Recombinant RNase Inhibitor, and 10 units/μl of SuperScript™ III RT in a final volume of 20 μl [35].

The third step was to identify the infection-causing enterovirus using the multiplex PCR technique. For a total reaction volume of 50 μL, 3 μL of synthesized first-strand cDNA was used as a template for PCR amplification with 1X PCR buffer (HotStarTaq DNA polymerase Kit, QIAGEN), 400 μM dNTPs, 0.4 μM enterovirus primers (SO222R, 5’-CIC CIG GIG GIA YRW ACA T-3′; SO224, 5’-GCI ATG YTI GGI ACI CAY RT-3′), 1X Q-Solution and 2.5 units HotStarTaq DNA polymerase [36]. PCR was conducted in a T100™ Thermal Cycler (Bio-Rad) and started with an initial denaturation step at 95 °C for 5 min followed by 40 cycles of 95 °C for 30 s, 48 °C for 30 s and 72 °C for 45 s, ending with a final extension step at 72 °C for 7 min.

Positive specimens were sent for detection in the fourth step. This step aimed to classify the EV-A71 or Coxsackievirus. A similar reaction was set up with different pairs of primers: 2 μM each CA16 primer (T79F, 5’-CCT ACM GCY GCC AAY ACY RAW GC-3′; T314R, 5’-CCA TCK GTG TTY TGK GTR CC-3′) or 2 μM each EV71 primer (T001F, 5’-ACY ATG AAA YTG TGC AAR G-3′; T002R, 5’-CCR GTR GGI GTR CAY GCA AC-3′) with the same annealing temperature of 48 °C.

In the final step, 92 specimens (4–5 cases from each episode of an outbreak) were purposively selected from the early cases (Case No.1st to case No. 5th) of each outbreak for genome identification by commercial automated DNA sequencing. The serotype was identified after serotyping by similarity searches and BLAST searches. Data from the questionnaires and laboratory results were merged for statistical analysis (Fig. 2).

### Statistical analysis

Microsoft Excel was used for double entry and data validation. Data were analyzed by SPSS version 24, 2016 (SPSS, Chicago, IL). Both descriptive and inferential statistics were used to describe the general characteristics of participants and to test the associations between variables. Chi-square test or Fisher’s exact test was used to detect the associations of clinical features with the serotype of HFMD. Logistic regression was used to identify the factors associated with HFMD. The “ENTER” mode was used to detect associations in both the univariate and multivariable steps. All significant variables in the univariate analysis were subsequently used in the multivariable analysis to establish the final fixed model. The alpha was set at 0.05 in both the univariate and multivariable analyses (Additional file 2).

## Results

In total, 3505 cases of HFMD from Chiang Rai Province and 1191 cases from Pha Yao Province were reported in 2016. Twenty-one outbreaks were reported through the public health surveillance system in these two provinces between January 1, 2016, and December 31, 2016: 13 outbreaks from Chiang Rai Province, and 8 outbreaks from Pha Yao Province. Seven hundred and eighty-one (781) cases of HFMD that were reported from 13 outbreaks in Chiang Rai Province accounted for 22.3% of reported cases in the entire year. Three hundred forty-two (342) cases reported from 8 outbreaks in Pha Yao Province accounted for 28.7% of the reported cases in the entire year. The incidence rates of HFMD were 279.72/100,000 person-years in Chiang Rai Province and 321.24/100,000 person-years in Pha Yao Province (Fig. 2).

Throat swabs were obtained for 612 out of 1123 patients (54.5%), and those patients were recruited into the study: 59.6% were from Chiang Rai Province (365 patients), 51.8% were ill in the rainy season, 57.2% were males, and the majority were under 2 years old (57.5%) and lived in rural areas (57.5%). The average time from the onset of the illness to the end of its course was 4.04 days (min = 2 days, max = 7 days); patients positive for EV-A71 had a longer duration of illness than other patients (mean 5.20 days), and the average number of days after the onset of disease before a visit to a doctor was 1.70 days (min = 1 day, max = 6 days). One-quarter of the patients had participated in a group activity before disease onset, and most patients presented to the hospitals as outpatients (86.9%). Almost half of the patients were breastfed by their mothers for less than 6 months (40.5%). Additionally, 8.2% of patients were vaccinated with an incomplete schedule, 4.6% had a family member who was less than 6 years old, and 12.7% had been diagnosed with HFMD in the previous year (Table 1).

**Table 1:** General characteristics of HFMD patients

Characteristic	*n*	%
Total	612	100.0
Province		
Chiang Rai	365	59.6
Pha Yao	247	40.4
Season		
Summer (November–February)	102	16.7
Rainy (June–October)	317	51.8
Cold (March–May)	193	31.5
Sex		
Male	350	57.2
Female	262	42.8
Age (years)		
< 2	352	57.5
2–3	146	23.9
> 3	114	18.6
Residential area		
Rural	352	57.5
Municipal	260	42.5
Participated in a group activity 2 weeks prior to disease onset		
No	138	22.6
Yes	474	77.4
Type of attendance in a hospital		
Outpatient	532	86.9
Inpatient	80	13.1
History of breastfeeding		
No breastfeeding	24	3.9
≤ 6 months	224	36.6
> 6 months	364	59.5
Vaccination history		
Complete and timely	562	91.8
Incomplete and/or not timely	50	8.2
Place of daytime care		
Home	204	33.3
Day care center	348	56.9
Children’s school	60	9.8
Congenital disease		
No	582	95.1
G6PD	16	2.6
Allergy	10	1.7
Thalassemia	2	0.3
Asthma	2	0.3
Number of family members (persons)		
< 4	342	55.9
4–7	218	35.6
> 7	52	8.5
Number of family members aged < 6 years old (persons)		
≤ 2	584	95.4
> 2	28	4.6
History of HFMD in the previous year		
No	534	87.3
Yes	78	12.7

Regarding the characteristics of parents, 88.9% were married, and 98.0% were Buddhist. Almost half of the fathers (49.4%) and 37.8% of mothers worked as a daily wage employee in an unprofessional work sector. More than half of the caregivers (57.5%) were uneducated or studied below the primary school level (Table 2).

**Table 2:** General characteristics of parents’ of HFMD patients

Characteristic	*n*	%
Total	612	100.0
Marital status		
Single	18	2.9
Married	544	88.9
Widow and divorce	50	8.2
Religion		
Buddhism	600	98.0
Christianity	12	2.0
Father’s occupation		
Unemployed	66	10.8
Government employee	24	3.9
Farmer	142	23.2
Daily-wage employee	302	49.4
Unprofessional skilled job	78	12.7
Mother’s occupation		
Unemployed	168	27.5
Government employee	18	2.9
Farmer	108	17.7
Daily-wage employee	232	37.8
Unprofessional skilled job	86	14.1
Major caregiver		
Father	14	2.3
Mother	346	56.5
Relative	202	33.0
Father and mother	50	8.2
Education of caregiver		
Not educated	174	28.4
Primary school	178	29.1
Secondary school	82	13.4
High school	108	17.7
Vocational and higher	70	11.4
Daily cleaning of food and drink containers		
No	32	5.2
Yes	580	94.8

In total, 612 patients with a diagnosis of HFMD were selected to undergo specimen collection for laboratory detection. Only 42.5% of those patients were positive for human enterovirus. Among patients who were positive for human enterovirus, 17.7% tested positive for coxsackievirus, 56.1% for EV-A71, and 26.2% for other human RNA enteroviruses.

Among 92 patients who were positive for human enterovirus, 4–5 patients from each outbreak were further analyzed to identify the serotype. The results indicated that 34.8% of patients were positive for EV-A71, 26.1% for CV-A16, 15.2% for CV-A6, 10.9% for CV-A10, and 2.2% for human enterovirus (Table 3).

**Table 3:** Serotype data of the HFMD patients

Type	*n*	%
Total	612	100.0
Human enterovirus species		
Negative	352	57.5
Positive	260	42.5
Serotype among positive		
EV-A*	146	56.1
Coxsackievirus**	46	17.7
Other human RNA enteroviruses	68	26.2
92 selected cases for serotyping		
CV-A10	10	10.9
CV-A16	24	26.1
CV-A4	2	2.2
CV-A6	14	15.2
CV-B2	2	2.2
EV-A71	32	34.8
Human rhinovirus	2	2.2
Unknown	6	6.4

*EV-A refers EV-A71

**Coxsackievirus refers CV-A10, CV-A16, CV-A4, CV-A6, CV-B2

In the multivariable analysis of the factors associated with positive enterovirus detection among the 612 patients, the following two variables had significant associations: history of breastfeeding and occupation of mother. Children who had never been breastfed and those with a history of breastfeeding ≤6 months had a 3.74-fold (95% CI = 1.53–9.10) and 2.52-fold (95% CI = 1.76–3.61) greater chance of suffering from EV-associated HFMD than children who had a history of breastfeeding > 6 months, respectively. Children who had mother who worked as farmers, daily wage employee, and unprofessional skilled job had a 1.88-fold (95% CI = 1.11–3.16), 2.18-fold (95% CI = 1.41–3.37), and 2.64-fold (95% CI = 1.52–4.61) greater chance of EV-associated HFMD than children who had mother as unemployed, respectively (Table 4).

**Table 4:** Univariate and multivariable analyses for the identification of factors associated with human enteroviruses among HFMD patients

Characteristic	Enterovirus		OR	95% CI	*p*-value	OR_adj_	95% CI	*p*-value
	n	(%)						
Sex								
Male	154	59.2	1.16	0.84–1.60	0.381			
Female	106	40.8	1.00					
Age (years)								
< 2	146	56.2	0.73	0.48–1.12	0.153			
2–3	58	22.3	0.68	0.42–1.12	0.130			
> 3	56	21.5	1.00					
Residential area								
Rural	150	57.7	1.00					
Municipal	110	42.3	0.99	0.71–1.37	0.940			
Participated in a group activity 2 weeks before disease onset								
No	66	25.4	0.76	0.52–1.11	0.150			
Yes	194	74.6	1.00					
Breastfeeding								
No	14	5.4	2.78	1.20–6.43	0.017	3.74	1.53–9.10	0.004*
≤ 6 months	124	47.7	2.46	1.75–3.46	< 0.001*	2.52	1.76 – 3.61	< 0.001*
> 6 months	122	46.9	1.00			1.00		
History of vaccination								
Complete and timely	242	93.1	1.00					
Incomplete or/and not timely	18	6.9	0.74	0.41–1.36	0.335			
Day time care								
Home	78	30.0	1.00					
Days care center	154	59.2	1.28	0.90–1.83	0.167			
School	28	10.8	1.41	0.79–2.53	0.243			
Congenital disease								
No	248	95.4	1.00					
Yes	12	4.6	0.90	0.43–1.90	0.778			
Regularly drug use								
No	254	97.7	1.00					
Yes	6	2.3	2.06	0.57–7.36	0.268			
Family member (persons)								
< 4	136	52.3	1.00					
4 - 7	98	37.7	1.24	0.88–1.74	0.225			
> 7	26	10.0	1.52	0.84–2.72	0.164			
Family members < 6 years old (persons)								
≤ 2	248	95.4	1.00					
> 2	12	4.6	1.02	0.47–2.19	0.967			
History of HFMD previous year								
No	228	87.7	1.00					
Yes	32	12.3	0.93	0.58–1.51	0.780			
Religion								
Buddhism	256	98.5	1.49	0.44–5.00	0.520			
Christianity	4	1.5	1.00					
Father’s occupation								
Unemployed	32	12.3	1.00					
Government employee	6	2.3	0.35	0.13–1.01	0.051			
armer	56	21.5	0.69	0.38–1.25	0.220			
Daily wage employee	132	50.8	0.83	0.48–1.41	0.480			
Other	34	13.1	0.82	0.43–1.59	0.557			
Mother’s occupation								
Unemployed	50	19.2	1.00			1.00		
Government employees	6	2.3	1.18	0.42–3.32	0.754	0.95	0.33–2.74	0.946
Farmer	46	17.7	1.75	1.06–2.90	0.030*	1.88	1.11 – 3.16	0.018*
Daily wage employee	114	43.9	2.28	1.50–3.47	< 0.001*	2.18	1.41 – 3.37	< 0.001*
Unprofessional skilled job	44	16.9	2.47	1.45–4.23	0.001*	2.64	1.52 – 4.61	0.001*
Major caregiver								
Father	10	3.9	1.00					
Mother	122	46.9	0.22	0.07–0.71	0.011*			
Relative	92	35.4	0.34	0.10–1.10	0.072			
Father and mother	36	13.8	1.03	0.28–3.83	0.966			
Marital status of caregiver								
Single	8	3.1	1.00					
Married	230	88.4	0.92	0.36–2.36	0.855			
Divorce	22	8.5	0.98	0.33–2.91	0.974			
Education level of caregiver								
Not educated	68	26.2	1.00					
Primary school	82	31.5	1.33	0.87–2.03	0.185			
Secondary school	34	13.1	1.10	0.65–1.88	0.716			
High school	32	12.3	0.65	0.39–1.10	0.108			
Vocational school or higher	44	16.9	2.64	1.49–4.68	0.001*			
Daily cleaning of food and drink containers								
No	6	2.3	1.00					
Yes	254	97.7	3.38	1.37–8.33	0.008*			

*Statistically significant at α=0.05

Five clinical features were associated with different serotypes among 576 cases (36 missing, accounted for 5.8%). Patients with coxsackievirus and other human enteroviruses had a greater chance of having a rash on their buttocks, knees, and elbows, having a fever, and sore throat or mouth ulcer than those with EV-A71. Those with coxsackievirus had a greater chance of experiencing lethargy and malaise than those with EV-A71 and other human enterovirus (Table 5).

**Table 5:** Associations of the serotype with the signs and symptoms of HFMD (*n* = 576 cases, missing 36 cases)

Signs and symptoms		Coxsackievirus *n* (%)	Enterovirus A71 *n* (%)	Other human enterovirus *n* (%)	Nonhuman enterovirus *n* (%)	χ^2^	*p*-value
Rash on palms and soles	Yes	36 (94.7)	114 (81.4)	52 (83.9)	288 (85.7)	4.437	0.218
	No	2 (5.3)	26 (18.6)	10 (16.1)	48 (14.3)		
Rash on buttocks, knee and elbow	Yes	14 (36.8)	20 (14.3)	26 (41.9)	106 (31.6)	22.026	< 0.001*
	No	24 (63.2)	120 (85.7)	36 (58.1)	230 (68.4)		
Fever	Yes	24 (63.2)	76 (54.3)	56 (90.3)	214 (63.7)	24.462	< 0.001*
	No	14 (36.8)	64 (45.7)	6 (9.67	122 (36.3)		
Sore throat or mouth ulcer	Yes	36 (94.7)	130 (92.9)	54 (87.1)	274 (81.6)	13.258	0.004*
	No	2 (5.3)	10 (7.1)	8 (12.9)	62 (18.4)		
Weakness	Yes	10 (26.3)	14 (10.0)	8 (12.9)	58 (17.3)	7.686	0.053
	No	28 (73.7)	126 (90.0)	54 (87.1)	278 (82.7)		
Anorexia	Yes	20 (52.6)	58 (41.4)	20 (32.3)	130 (38.7)	4.408	0.221
	No	18 (47.4)	82 (58.6)	42 (67.7)	206 (61.3)		
Diarrhea	Yes	2 (5.3)	6 (4.3)	2 (3.2)	32 (9.5)	5.452	0.129^a^
	No	36 (94.7)	134 (95.7)	60 (96.8)	304 (90.5)		
Lethargy	Yes	10 (26.3)	10 (7.1)	8 (12.9)	30 (8.9)	13.433	0.004*
	No	28 (73.7)	130 (92.9)	54 (87.1)	306 (91.1)		
Malaise	Yes	6 (15.8)	4 (2.9)	6 (9.7)	6 (1.8)	18.967	< 0.001*^,a^
	No	32 (84.2)	136 (97.1)	56 (90.3)	330 (98.2)		
Vomiting	Yes	4 (10.5)	4 (2.9)	4 (6.5)	24 (7.1)	4.855	0.168^a^
	No	34 (89.5)	136 (97.1)	58 (93.5)	312 (92.9)		

*Statistically significant at α = 0.05

^a^Fisher’s exact test

## Discussion

Children less than 2 years old are the major vulnerable population for HFMD infection in northern Thailand. However, the proportion of diagnosed HFMD cases was not significantly different between sexes. The rainy season is the epidemic period during the year. The incidence rates in northern Thailand are higher than the Thai national rate and are also higher than other regions in the Asia-Pacific Region, such as Vietnam [37], Taiwan [38], China [39], and Hong Kong [40]. Based on these reports, more than half of HFMD cases are the EV-A serotype. A closer look into the sub-serotype in 92 selected cases showed that EV-A71 was dominant in the enterovirus group, and CV-A16 was dominant in the coxsackievirus group. The average duration from disease onset to the end of its course was 4.04 days, and the average number of days after the onset of disease before visiting a doctor was 1.70 days. Most HFMD patients attended the outpatient department. A history of breastfeeding ≤6 months and the occupation of the parents were associated with enterovirus HFMD infection among children. Children who had mother who worked as farmers, daily wage employee, and unprofessional skilled job had greater chance of EV-associated HFMD than children who had mother as unemployed. Patients with coxsackievirus and other human enteroviruses had a greater chance of having a rash on their buttocks than those with EV-A71. Patients with coxsackievirus had a greater chance of experiencing lethargy and malaise than those with EV-A71 and other human enterovirus.

In our study, most of the infected persons were children aged less than 2 years old, which is consistent with the study by Rao et al. [41] that presented the most infected persons with HFMD in India. Wang et al. [42] also supported that children less than 2 years old were the most vulnerable population for HFMD in China. Regarding sex, in our study, more than half of the patients were male (57.4%), which is similar to the study by Zou et al. [43], which reported that most of the HFMD-infected children in China were male, accounting for 60.0% of all cases.

Inta et al. [29] and Upala et al. [30] reported that the specific climate characteristics of the northern region of Thailand, particularly the temperature, moisture, air pressure, and rainfall amount, contributed to the epidemic of HFMD. In our study, the highest number of HFMD episodes was reported in the rainy season (June–October). Xu et al. [44] found that environmental temperature was the key contributing factor to the number of HFMD cases in southern China.

Suliman et al. [45] reported that the occupation and behaviors of parents were associated with HFMD infection among preschool children. This finding is consistent with our study, which found that children with parents in some occupations accounted for a greater proportion of children with diagnoses of HFMD than those with parents in other occupations.

Many studies have reported that human enteroviruses classified from HFMD patients have different serotype proportions. In our study, we found that 56.1% of detected pathogens were EV-A71, and 17.7% were coxsackievirus. This finding was consistent with that of the study by He et al. [46], who reported that EV-A71 accounted for 41.9% of HFMD cases among young children in southern China, whereas a study in Guangdong, China, reported that only 22.4% of HFMD cases were classified as EV-A71 positive, and 23.0% were coxsackievirus positive [47]. Interestingly, Li et al. [48] reported that CV-A6 played a dominant role in several outbreaks in China and many other countries in Asia. Our study also found that 15.2% of HFMD cases were CV-A6 positive.

In this study, we found that 42.5% of patients with a diagnosis of HFMD were positive for human enterovirus, but He, et al. [46] reported that almost 91.3% of cases were positive for human enterovirus. A study in Shanghai reported that among 614 HFMD patients, only 19.9% were EV-A71 positive [47]. Ganorkar et al. [6] reported that the main serotype of CV among HFMD patients was CV-A16. This finding was consistent with our study, which showed that CV-A16 was the main sub-serotype followed by CV-A6.

Inta et al. [29] reported that having a history of breastfeeding for more than 6 months was a protective factor for HFMD in school children in Thailand. The study in Vietnam also reported that socioeconomic status, including breastfeeding of infants, was a protective factor for HFMD [37]. Moreover, a neonatal mouse model used to assess the effectiveness of breastfeeding found that breastfeeding played a protective role in HFMD, which could be due to the IgG acquired from mothers during breastfeeding [49]. This finding is consistent with our study, which found that children with no history of breastfeeding and those who breastfed for less than 6 months had a greater chance of acquiring an HFMD infection.

Wei et al. [50] reported that patients with EV-A71 infection had a longer duration of high fevers and a longer duration of illness than patients with HFMD of other causes in China. Zou [43] reported that EV-A71 was associated with severe cases of HFMD in Guangdong, China. In contrast, our study found that some clinical features, such as rash, mouth ulcers, and fever, are related to coxsackieviruses. A prospective cohort study conducted in Cambodia reported that EV-A71 was associated with severe clinical signs and symptoms among HFMD-infected children, particularly those with nervous system complications [51]. EV-A71 is a predominant serotype of an HFMD outbreak in Spain and is associated with severe clinical signs such as brainstem symptoms [52]. A study in China reported that the severity of HFMD is not only impacted by the virus serotype but also the age and comorbidity of patients [45]. Parental behaviors are also associated with the severity of the disease [45]_._ Ooi et al. reported that most severe cases of HFMD were caused by EVA-71 [53].

Some limitations in the study may affect the study results. Thirteen out of ninety-six specimens that were collected from the early cases of HFMD outbreak were stored for more than 24 h at the original institute before reaching the laboratory due to the distance between the hospitals and the laboratory. The working hours of the laboratory are only Monday to Friday, but outbreaks sometimes occurred during the weekend. The researchers addressed this problem by initiating a proper storage procedure in the hospital to maintain the quality of the specimens.

In our study, the laboratory was in Chiang Rai Province. Therefore, the specimens obtained from hospitals in Pha Yao Province were difficult to transport on time. Additionally, seven parents could not remember whether their children were previously diagnosed with HFMD, and four parents could not provide an accurate history of congenital disease in their children during the interview. Finally, this information was obtained from medical records after acquiring approval from the parents and the doctors. However, participants who responded with “no previous diagnosis of HFMD” may have introduced recall bias, as the medical records of those participants were not investigated. And 36 cases (5.8%) of the participants could not obtain the information regarding the clinical signs and symptoms. However, the missed data might not impact the analysis since there were a few number (only 5.8%), and the characteristics of missing data similar with those remaining in the analysis.

In addition, only 9 hospitals have a pediatric department. Therefore, specimen collection in the remaining 5 hospitals was completed by medical technicians, who are healthcare professionals and have completed a four-year university program focusing on training in laboratory work, including specimen collection from patients.

Another potential limitation in this study is selection bias, because some members of the study population did not meet the criteria to access healthcare services free of charge. Individuals with a Thai ID card were more likely to access medical care than those without a Thai ID card. The selection bias may extend to other populations with specific characteristics. For instance, parents of children aged 2–4 years tended to seek medical care more often than other individuals [54].

Last, we used throat swabs to collect specimens for detection in the laboratory instead of stool. During the pilot test, we collected stool samples, but we found that this method was not effective in young children, as specimens were difficult to obtain.

## Conclusion

Northern Thailand is presented as an epidemic area of HFMD particularly in Chiang Rai and Pha Yao provinces. A number of infected persons have been reported through several episodes of HFMD outbreak. EV-A71, CV-A16, and CV-A6 are major causes of HFMD in children < 6 years. Less of breastfeeding time and unprofessional job of their mother are the factors associated with HFMD in their children. Patients with coxsackievirus and other human enteroviruses have a variety of clinical signs and symptoms than those with EV-A71. All children should be provided in breastfeeding from their mother at least 6 months after getting birth. Public health interventions should be promoted for mothers in child caring skills to prevent HFMD particularly those who are working unprofessional job sectors.

## Additional files

Additional file 1: Questionnaire. It is a set of questions which was used for collecting data in the project. It was developed by authors, and never been published elsewhere. (PDF 89 kb)

Additional file 2: HFMD_Data t is the coded data of the HFMD project which was conducted in northern Thailand. (XLSX 233 kb)

## Abbreviations

CV: Coxsackievirus
DCC: Day care center
EPI: Expanded program of Immunization
EV: Enterovirus
G-6-PD: Glucose-6-phosphate dehydrogenase deficiency
HFMD: Hand, foot and mouth disease
ICD-10: International classification of disease version 2010
ID: Identification card
IgG: Immunoglobulin G
IOC: Item-objective congruence
IPD: Inpatient department
OPD: Outpatient department
PCR: Polymerase chain reaction
RT-PCR: Reverse transcriptase polymerase chain reaction
SD: Standard deviation
WHO: World Health Organization
