# Supplementary material for: Molecular epidemiology and clinical features of hand, foot and mouth disease in northern Thailand in 2016: a prospective cohort study
Source: BMC Infect Dis. 2018 Dec 6;18:630. doi: 10.1186/s12879-018-3560-4 (PMC6282397; doi:10.1186/s12879-018-3560-4)
Supplement: Supplementary file 1 — Questionnaire. It is a set of questions which was used for collecting data in the project. It was developed by authors, and never been published elsewhere. (PDF 89 kb) [file 12879_2018_3560_MOESM1_ESM.pdf]

ID……….

□ CASE (Medical diagnosis) □ CASE (Clinical Signs) □ CONTROL

Questionnaire for hand foot mouth project

Part 1 Children's information

1.1 Sex □ Male □ Female
1.2 Age ....year...........month
1.3 Weight..................................kg
1.4 Height ....cm
1.5 Resident area □ Municipal □ Rural
1.6 Group activity two weeks prior □ Yes □ No
1.7 Place of birth □ Hospital □ Home □ Other….
1.8 History of vaccination □ Complete and on-time □ Complete but some delay □ Incomplete
1.9 History of breastfeeding
□ No □ ≤ 3 months □ 3-6 months □ More than 6 months
1.10 Place of caring □ Home □ Day care center □ School
1.11 Underlying disease □ No □ Yes, specify.................................
1.12 Taking drug everyday □ No □ Yes, specify.................................
1.13 Family member..........person, and number of people aged less than 6 years…. person
1.14 Having HFMD previously □ No □ Yes, year……….

Part 2 Parents' information

2.1 marital status
□ Single □ Married □ Divorced □ other…..
2.2 Religion
□ Buddhist □ Christ □ Islam □ Other, specify..............
2.3 Father's occupation □ Unemployed □ Officer □ Agriculturist □ Employed □ Trader □ Other, specify……..Income................../month
2.4 Mother's occupation □ Unemployed □ Officer □ Agriculturist □ Employed □ Trader □ Other, specify……..Income................../month

Part 3 Care giver's information

3.1 Major care giver □ Father □ Mother □ Relatives
3.2 Education
□ None □ Primary □ Secondary
□ High school □ Vocational □ University
3.3 Cleaning utensil for food storage
□ No □ Yesทำ (□ by water □ by solvent)
3.4 Major handle for all expenses regarding child caring (Possible to choose more than one choice)
□ Father □ Mother □ Relatives

Part 4 Medical history of child

4.1 Initial date of illness ....
4.2 Date visit a doctor.....................................
□ Not visit a doctor □ Public hospital □ Private clinic

4.3 Sign and symptoms

Sign and symptoms	Present		Day						
	Yes	No	1	2	3	4	5	6	7
Rash on palms and soles									
Rash on buttocks, knee and elbow									
Fever									
Sore throat or mouth ulcer									
Weak									
Anorexia									
Diarrhea									
Shock									
Headache									
Shabby									
Convulsion									
Coma									
Stop breathing									
Blood pressure reducing									
Vomiting									
Cough with blood									
